# Einarbeitung in der Intensiv- und Notfallmedizin in Deutschland

**DOI:** 10.1007/s00063-024-01108-0

**Published:** 2024-02-02

**Authors:** Jan-Hendrik Naendrup, Anna Carola Hertrich, Janika Briegel, Eyleen Reifarth, Julian Hoffmann, Anuschka Mucha, Victoria König, Theresa Weber

**Affiliations:** 1 Klinik I für Innere Medizin, Centrum für integrierte Onkologie Aachen Bonn Köln Düsseldorf, Medizinische Fakultät und Uniklinik Köln, Universität zu Köln, Kerpener Str. 62, 50937 Köln, Deutschland; 2 Zentrum für Anästhesiologie und Intensivmedizin, Klinik für Intensivmedizin, Universitätsklinikum Hamburg-Eppendorf (UKE), Hamburg, Deutschland; 3 Innere Medizin III – Kardiologie und Angiologie, Universitätsklinikum Tübingen, Tübingen, Deutschland; 4 Klinik für Innere Medizin 8, Schwerpunkt Kardiologie, Abteilung für Intensivmedizin, Klinikum Nürnberg, Nürnberg, Deutschland; 5 Hamburg, Deutschland

**Keywords:** Fortbildung, Weiterbildung, Intensivstation, Notaufnahme, Initial training, Further education, Intensive care unit, Emergency department

## Abstract

**Hintergrund:**

Die Therapie akut lebensbedrohlicher Krankheitsverläufe in der Intensiv- und Notfallmedizin verlangt nach einer fundierten Aus- und Weiterbildung, wobei der Einarbeitung eine Schlüsselrolle zufällt.

**Fragestellung:**

Wie sind die Struktur und Qualität der ärztlichen und pflegerischen Einarbeitung auf Intensivstationen und in Notaufnahmen in Deutschland?

**Methodik:**

Mithilfe einer Befragungssoftware wurde ein deutschsprachiger Online-Fragebogen mit 40 Fragen zur genannten Thematik zur anonymisierten Datenerhebung entworfen. Die Verbreitung erfolgte über die Website der Deutschen Gesellschaft für Internistische Intensivmedizin und Notfallmedizin (DGIIN) und über Social-Media-Kanäle.

**Ergebnisse:**

103 Pflegefachpersonen und 125 Ärzt:innen nahmen an der Befragung teil. Die Berufserfahrung der Pflegefachpersonen lag bei durchschnittlich 8,5 ± 5,1 Jahren, die der Ärzt:innen bei 3,0 ± 3,1 Jahren. Die Teilnehmenden arbeiteten schwerpunktmäßig auf Intensivstationen (59 %) oder in Notaufnahmen (22 %). Die Einarbeitung der Pflegefachpersonen dauerte durchschnittlich 45 ± 27 Tage, die der Ärzt:innen 13 ± 13 Tage. Nur 20 % der Einarbeitung erfolgten losgelöst von der klinischen Routineversorgung als Seminar oder Praxistraining. 47 % der Teilnehmenden gaben an, dass sie die avisierte Einarbeitungszeit nicht vollständig absolvieren konnten. Nur 49 % wurden rechtskonform in die Geräte ihres Arbeitsbereichs eingewiesen. Nach der Einarbeitung gaben 35 % an, sich sicher oder eher sicher im Umgang mit planbaren Tätigkeiten zu fühlen, bei akuten Notfällen waren es 15 %.

**Diskussion:**

Die Einarbeitung in der Intensiv- und Notfallmedizin ist vielerorts inadäquat und birgt sowohl Sicherheits- als auch Haftungsrisiken. Es bedarf neuer Konzepte, um die Einarbeitung klinikübergreifend zu verbessern.

**Zusatzmaterial online:**

Zusätzliche Informationen sind in der Online-Version dieses Artikels (10.1007/s00063-024-01108-0) enthalten.

## Abstract (en)

**Background:**

The treatment of acute life-threatening conditions in intensive care and emergency medicine requires in-depth training and education, with initial training playing a key role.

**Research question:**

What is the structure and quality of the initial training of physicians and nurses in intensive care units and emergency departments in Germany?

**Methods:**

With the help of survey software, an online questionnaire comprising 40 questions in German on the aforementioned topic was designed. It was distributed via the website of the German Society for Internal Intensive Care and Emergency Medicine (DGIIN) and via pertinent social media channels.

**Results:**

In total, 103 nurses and 125 physicians participated in the survey. The average work experience of the participating nurses and physicians was 8.5 ± 5.1 and 3.0 ± 3.1 years, respectively. The majority of participants worked primarily in intensive care units (59%) or in emergency departments (22%). On average, the initial training lasted 45 ± 27 and 13 ± 13 days for nurses and physicians, respectively. Only 20% of the initial training comprised seminars or hands-on workshops taught outside of routine clinical care. In all, 47% of the participants stated that they were not able to complete the entire initial training period. Only 49% had been officially certified for usage of the technical equipment in their department. A total of 35% reported feeling confident or somewhat confident in handling predictable tasks after initial training, but only 15% in handling acute emergencies.

**Discussion:**

The present study revealed that initial training in intensive care and emergency medicine is frequently incomplete, unstructured, as well as inadequate and bears both safety and liability risks. New concepts are needed to improve the initial training across clinics.

Um den hohen Anforderungen in der Intensiv- und Notfallmedizin gerecht zu werden, muss allen beteiligten Berufsgruppen eine qualifizierte Weiterbildung zuteilwerden. Dabei kommt der Einarbeitung zu Beginn der Tätigkeit auf der Intensivstation bzw. in der Notaufnahme eine besondere Bedeutung zu: Eine umfassende, systematische Einarbeitung legt das Fundament für eine hochwertige, fachgerechte Ausbildung und letztlich auch für eine hochwertige, sichere und professionelle Patient:innenversorgung.

## Einleitung

Die Therapie schwerstkranker Menschen steht im Mittelpunkt der Intensiv- und Notfallmedizin. Heutzutage sehen sich diese Disziplinen durch den demografischen Wandel mit einer stetig wachsenden Zahl an älteren und multimorbiden Patient:innen konfrontiert [6, 12]. Gleichzeitig resultieren aus stetigem methodischem und technischem Fortschritt immer komplexere Behandlungsstrategien, deren Implementierung sowohl umfangreiches theoretisches Wissen als auch praktische Kompetenzen voraussetzt [1, 3, 16].

Damit Schwersterkrankte stets die bestmögliche Therapie nach neustem Erkenntnisstand erfahren, müssen interdisziplinäre und multiprofessionelle Behandlungsteams auf hohem fachlichem Niveau zusammenarbeiten. Diese Behandlungsteams setzen sich neben dem ärztlichen Personal und Pflegefachpersonen auch aus Rettungsassistent:innen und Notfallsanitäter:innen zusammen. Auf der Intensivstation werden diese Teams noch um weitere Mitarbeitende aus Physiotherapie, Atmungstherapie und Ergotherapie sowie Logopädie und Pharmazie ergänzt. Zusätzlich etablieren sich sowohl in der Notaufnahme als auch auf der Intensivstation zunehmend die Berufsbilder der Physician Assistants (Pas) sowie Advanced Practice Nurses (APN). Alle am Behandlungsprozess Beteiligten müssen über ein hohes Maß an fachlichen sowie sozialen Kompetenzen verfügen, um ihre jeweilige Rolle zu erfüllen und gemeinsam das angestrebte Behandlungsziel zu erreichen.

Um diesen hohen Anforderungen in der Intensiv- und Notfallmedizin gerecht zu werden, bedarf es einer qualifizierten Fort- und Weiterbildung aller Berufsgruppen. Hierbei fällt der Einarbeitung eine besondere Bedeutung zu, da hier das Fundament für ein sicheres und fachgerechtes Arbeiten gelegt wird.

Das Ziel der vorliegenden Umfrage war es, zu erfassen, wie sich für ärztliches und pflegerisches Personal der Einstieg in den Arbeitsalltag auf deutschen Intensivstationen bzw. in Notaufnahmen gestaltet. Neben Punkten wie Dauer und praktischem Ablauf der Einarbeitungsphase wurden auch Aspekte wie individuelles Sicherheitsgefühl bei der späteren Durchführung von praktischen Tätigkeiten und subjektive Zufriedenheit im Arbeitsalltag abgefragt.

## Methodik

Mithilfe einer cloudbasierten Befragungssoftware (https://www.limesurvey.org/, LimeSurvey GmbH, Hamburg, Deutschland) wurde ein deutschsprachiger Online-Fragebogen zur Einarbeitung und Fortbildung auf Intensivstationen und in Notaufnahmen entworfen. Der Link zum Fragebogen wurde über die Website der Deutschen Gesellschaft für Internistische Intensivmedizin und Notfallmedizin (DGIIN), den DGIIN-Newsletter sowie die Social-Media-Kanäle der DGIIN verbreitet. Aufgrund dieser Verbreitung des Fragebogens bleiben die Gesamtzahl der Empfänger:innen sowie die tatsächliche Rücklaufquote unklar.

Die Umfrage umfasste 40 Fragen zur Struktur und Gestaltung der Einarbeitung, zur Regelmäßigkeit von Fortbildungen sowie zum Sicherheitsgefühl bei anschließenden Tätigkeiten. Die Antworten wurden anonym erhoben, sodass ein Rückschluss auf teilnehmende Kliniken oder konkrete Personen ausgeschlossen war.

Die Daten wurden mit Microsoft Excel (Version 16.74, Microsoft Corporation, Redmond, USA) und SPSS (Version 29, IBM, Armonk, USA) statistisch ausgewertet. Kontinuierliche Variablen wurden als Mittelwert ± Standardabweichung berichtet und mittels T‑Test verglichen, dichotome Variablen wurden als prozentualer Anteil der abgegebenen Antworten berichtet und mittels Chi-Quadrat-Test verglichen. Im Falle fehlender Werte erfolgte ein paarweiser Fallausschluss. Da es sich bei dieser Studie um eine freiwillige Umfrage handelte, war keine Zustimmung durch die örtlichen Ethikkommissionen erforderlich.

## Ergebnisse

### Teilnehmende

Der Link zur Umfrage wurde insgesamt 257-mal geöffnet; 103 Pflegefachpersonen und 125 Ärzt:innen begannen mit der Bearbeitung des Fragebogens. Die interprofessionellen Fragen wurden im Durchschnitt von 188 Personen beantwortet.

**Abb. 1:**
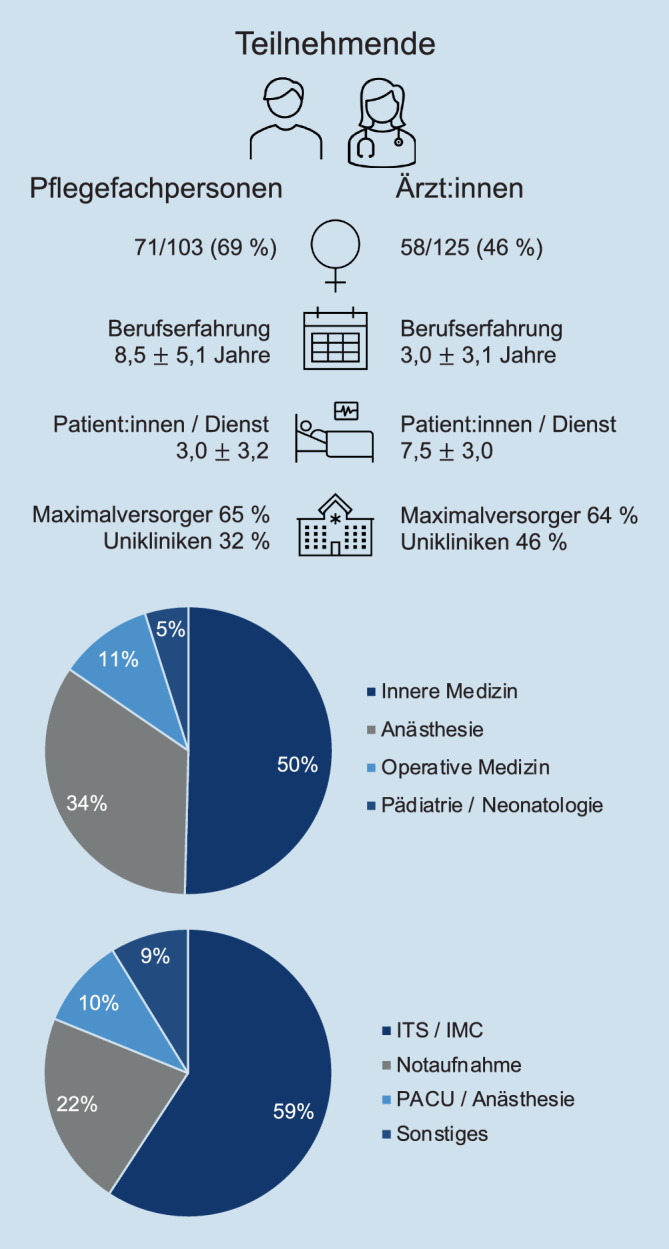
Demografische Übersicht der teilnehmenden Pflegefachpersonen und Ärzt:innen

Eine deskriptive Übersicht über die teilnehmenden Pflegefachpersonen und Ärzt:innen ist in Abb. 1 dargestellt. 57 % der Teilnehmenden waren weiblich, 43 % männlich. Insgesamt hatten die Teilnehmenden 4,9 (± 5,2) Jahre Berufserfahrung im Bereich Intensiv- und Notfallmedizin; 65 % arbeiteten an Krankenhäusern der Maximalversorgung, 40 % an Unikliniken. 50 % der teilnehmenden Pflegefachpersonen und Ärzt:innen arbeiteten im Fachbereich Innere Medizin, 34 % in der Anästhesiologie und 11 % in operativen Fachrichtungen. Dabei waren 59 % auf einer Intensiv- oder IMC-Station beschäftigt, 22 % in der Notaufnahme und 11 % im OP-Bereich bzw. der „post anesthesia care unit“ (PACU).

Die Pflegefachpersonen hatten durchschnittlich 8,5 (± 5,1) Jahre Berufserfahrung und zu 53 % eine 3‑jährige Ausbildung mit staatlichem Examen absolviert; 25 % hatten eine Fachweiterbildung für Anästhesie und Intensivmedizin. Ärztliches Personal hatte durchschnittlich 3,0 (± 3,4) Jahre Berufserfahrung. 72 % der Teilnehmenden waren Assistenzärzt:innen, 22 % waren Fachärzt:innen; 11 % hatten die Zusatzweiterbildung Intensivmedizin, 40 % die Zusatzweiterbildung Notfallmedizin.

### Einarbeitung

**Abb. 2:**
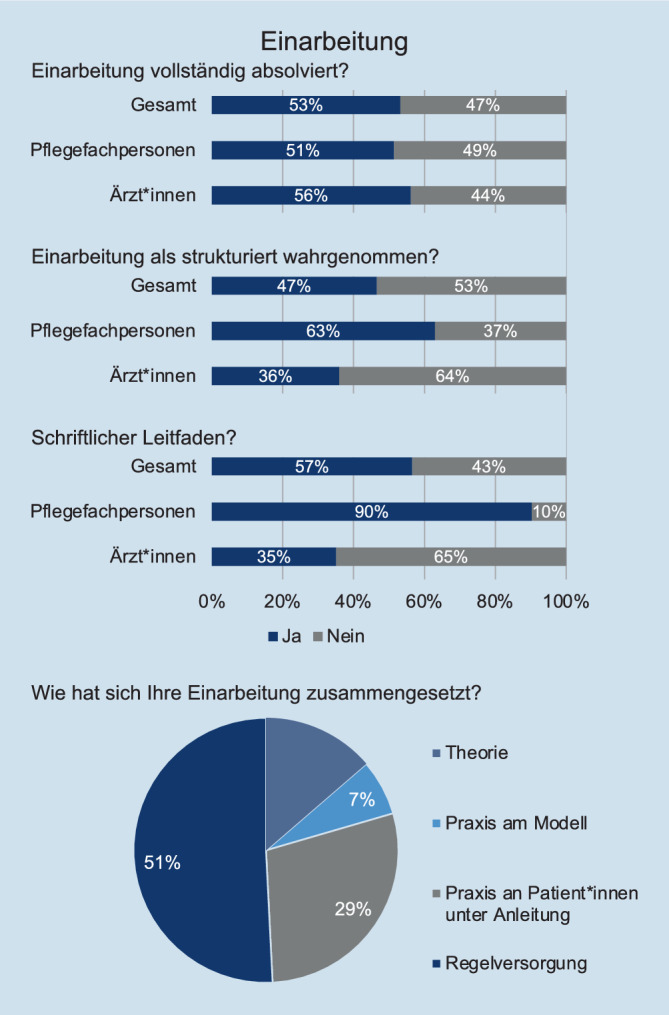
Vollständigkeit und Struktur der Einarbeitung von Pflegefachpersonen und Ärzt:innen

Eine Übersicht grundlegender Ergebnisse zur Einarbeitung ist in Abb. 2 dargestellt. Die Einarbeitung bei Pflegefachpersonen dauerte durchschnittlich 45 (± 27) Tage, die Einarbeitung des ärztlichen Personals durchschnittlich 13 (± 13) Tage. 51 % der Einarbeitung erfolgte als Regelversorgung von Patient:innen, 29 % in Form praktischer Tätigkeiten an Patient:innen unter Anleitung. 20 % waren von der klinischen Versorgung losgelöste Lehrveranstaltungen, 13 % in Form von Theorie und 7 % in Form von Praxistrainings am Modell. 47 % der Teilnehmenden gaben an, dass sie die initial angestrebte Einarbeitungszeit nicht vollständig absolvieren konnten, wobei es keine signifikanten Unterschiede zwischen Pflegefachpersonen und ärztlichem Personal gab (*p* = 0,554). 53 % der Teilnehmenden empfanden ihre Einarbeitung als strukturiert, wobei der Anteil beim ärztlichem Personal (36 %) gegenüber Pflegefachpersonen (63 %) signifikant geringer war (*p* = 0,001). Weiterhin stand ein schriftlicher Leitfaden Ärzt:innen (35 %) seltener zur Verfügung als Pflegefachpersonen (90 %, *p* < 0,001).

49 % der Teilnehmenden wurden rechtskonform in die Geräte in ihrem Arbeitsbereich eingewiesen, wobei 42 % der Teilnehmenden ihre Geräteinweisung als strukturiert wahrnahmen. Pflegefachpersonen waren dabei signifikant häufiger rechtskonform in Geräte eingewiesen als Ärzt:innen (62 % vs. 40 %, *p* = 0,004). 60 % des ärztlichen Personals nahmen externe Fortbildungsangebote zur Einarbeitung, z. B. ICU-Beginner-Kurse, in Anspruch, wobei nur in 48 % der Fälle die Kosten vom Arbeitgeber übernommen wurden.

### Fortbildung & Feedback

**Abb. 3:**
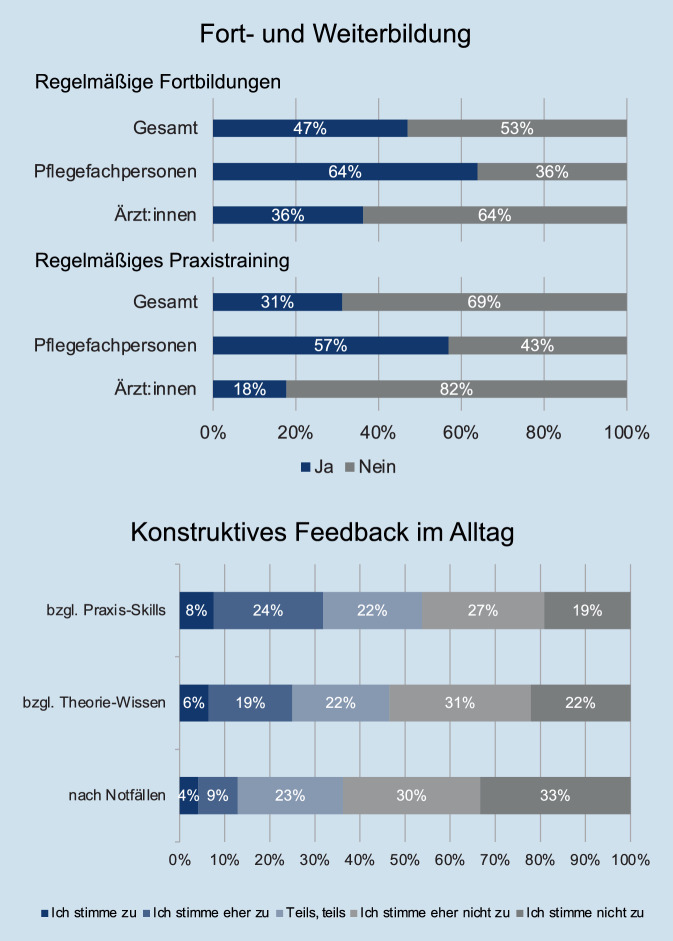
Fortbildung und Feedback im klinischen Alltag von Pflegefachpersonen und Ärzt:innen

Eine Übersicht grundlegender Ergebnisse zur Fortbildung und Feedbackkultur ist in Abb. 3 dargestellt. Regelmäßige Fortbildungen im Bereich der Akut- und Intensivmedizin fanden bei 47 % der Teilnehmenden statt. Dabei war das Angebot für Pflegefachpersonen signifikant größer als das für Ärzt:innen (64 % vs. 36 %, *p* < 0,001). Wenn Fortbildungen angeboten wurden, waren diese in 35 % ein- oder zweiwöchentlich, in 41 % monatlich und in 24 % seltener als einmal monatlich. Regelmäßige Praxisübungen mit dem Training von Notfallsituationen fanden bei 31 % der Teilnehmenden statt, wobei der Anteil bei Pflegefachpersonen (57 %) signifikant höher als bei Ärzt:innen (18 %) war (*p* < 0,001).

Aus dem klinischen Alltag gaben die Teilnehmenden an, dass konstruktives bzw. eher konstruktives Feedback in 25 % hinsichtlich ihres theoretischen Wissens und in 32 % hinsichtlich ihrer praktischen Fertigkeiten erfolgen würde. 64 % der Teilnehmenden berichteten, kein oder eher kein konstruktives Feedback nach akuten Notfällen zu erhalten.

### Sicherheitsgefühl & Belastung

Nach erfolgter Einarbeitung gaben lediglich 35 % der teilnehmenden Pflegefachpersonen und des ärztlichen Personals an, sich sicher oder eher sicher im Umgang mit planbaren Tätigkeiten zu fühlen. Bei der Versorgung akuter Notfälle berichteten 15 %, sich sicher oder eher sicher gefühlt zu haben; 58 % fühlten sich unsicher oder eher unsicher (vgl. Zusatz-Abb. 4). 53 % der Teilnehmenden gaben an, in solchen Fällen unkompliziert Hilfe von Fach- oder Oberärzt:innen einholen zu können; 19 % berichteten, dies nicht zu können. Pflegefachpersonen mit vollständig absolvierter Einarbeitung hatten ein signifikant höheres Sicherheitsgefühl bei planbaren Tätigkeiten im Vergleich zu Teilnehmenden ohne vollständige Einarbeitung (*p* = 0,048; bei Ärzt:innen *p* = 0,140), Ärzt:innen mit vollständig absolvierter Einarbeitung hatten dagegen ein signifikant höheres Sicherheitsgefühl bei der Versorgung akuter Notfälle (*p* = 0,008; Pflegefachpersonen *p* = 0,687). Im Gegensatz zu Pflegefachpersonen hatten teilnehmende Ärzt:innen, die ihre Einarbeitung als strukturiert wahrnahmen, ein signifikant höheres subjektives Sicherheitsgefühl bei planbaren Tätigkeiten (*p* = 0,013; bei Pflegefachpersonen *p* = 0,82) und Notfällen (*p* < 0,001; bei Pflegefachpersonen *p* = 0,52). Pflegefachpersonen hingegen zeigten ein signifikant höheres subjektives Sicherheitsgefühl bei planbaren Tätigkeiten, wenn ihnen ein Skript zur Verfügung stand (*p* = 0,029; bei Ärzt:innen *p* = 0,738). Ärzt:innen, die regelmäßig Praxistrainings absolvierten, hatten ein signifikant höheres Sicherheitsgefühl bei akuten Notfällen als Ärzt:innen ohne regelmäßiges Praxistraining (*p* = 0,010; bei Pflegefachpersonen *p* = 0,242); auf planbare Tätigkeiten zeigte sich keine Auswirkung.

46 % der Teilnehmenden gaben an, sich durch die Arbeit psychisch belastet zu fühlen. Dabei war die psychische Belastung bei Teilnehmenden, die eine als strukturiert wahrgenommene Einarbeitung erfuhren, signifikant geringer (*p* < 0,001). 26 % der Teilnehmenden berichteten, dass ihnen in persönlichen Gesprächen individuelle Ziele und Entwicklungsmöglichkeiten im Bereich der Akut- und Intensivmedizin aufgezeigt werden. Dabei fanden bei Ärzt:innen (16 %) diese Gespräche seltener als bei Pflegefachpersonen (42 %) statt (*p* = 0,005). Lediglich 35 % der Teilnehmenden, 41 % der Pflegefachpersonen und 31 % der Ärzt:innen, gaben an, dass sie sich vorstellen könnten, langfristig in der Akut- und Intensivmedizin zu arbeiten.

## Diskussion

Die Therapie akut lebensbedrohlicher Krankheitszustände in der Intensiv- und Notfallmedizin verlangt nach einer fundierten Aus- und Weiterbildung. Der erste Schritt zu einer erfolgreichen Ausbildung ist dabei eine strukturierte Einarbeitung. Die hier durchgeführte Umfrage konnte dabei aufzeigen, dass es einen subjektiv über alle Berufsgruppen hinweg empfundenen Mangel hinsichtlich der Qualität und Struktur der Einarbeitung in der Intensiv- und Notfallmedizin gibt. Es ist jedoch unerlässlich, in diesen hochkomplexen Bereichen eine fundierte Aus- und Weiterbildung, beginnend mit der Einarbeitung, zu erhalten [8, 17].

Verschiedene Studien, wie zuletzt die Untersuchung von Spacek et al., konnten belegen, dass sich ein standardisiertes Einarbeitungskonzept positiv auf die Versorgungsqualität und Patient:innensicherheit auswirkt [10, 17].

Trotz des nachweislichen Nutzens scheint es dennoch nicht die Regel zu sein, dass Kliniken in Deutschland über strukturiert konzipierte und didaktisch erfolgreiche Einarbeitungskonzepte für neue Beschäftigte verfügen. So gaben in unserer Umfrage nur ungefähr die Hälfte der Teilnehmenden an, dass sie ihre Einarbeitung als strukturiert empfunden hätten.

Zudem konnten knapp die Hälfte der Untersuchungsteilnehmenden ihre Einarbeitungszeit nicht vollständig absolvieren. Da die Umfrage die Gründe hierfür nicht erfragte, kann über mögliche Ursachen wie die Dynamik des Alltags in der Notfall- und Intensivmedizin oder mangelnde personelle Ressourcen nur gemutmaßt werden.

In diesem Zusammenhang sei auch darauf hingewiesen, dass gemäß Strukturempfehlung der DIVI Personal zu Beginn des Einsatzes auf einer Intensivstation eine Einarbeitungszeit von drei Monaten gewährt werden soll, in der die neuen Beschäftigten im Stellenschlüssel nicht berücksichtigt und zusätzlich zur regulären Personalausstattung eingeteilt werden sollten [5]. Diese drei Monate Einarbeitungszeit sind allerdings im klinischen Alltag aus strukturellen und monetären Gründen kaum umsetzbar; die durchschnittliche Einarbeitungsdauer im ärztlichen Bereich betrug unseren Ergebnissen zufolge lediglich zwei Wochen. Das pflegerische Personal war diesbezüglich etwas besser aufgestellt, doch auch hier lag die durchschnittliche Einarbeitungszeit von sechs Wochen deutlich unter der von der DIVI empfohlenen Zeitspanne.

Hinsichtlich der Ergebnisqualität der Einarbeitungsprogramme fühlten sich nach Abschluss der Einarbeitungsperiode nur 35 % der Befragten sicher im Umgang mit planbaren Tätigkeiten. Dieses Ergebnis ist insofern frappierend, als es gerade die planbaren Tätigkeiten sind, die während der Einarbeitung unter Supervision trainiert werden sollten. Für Notfallsituationen waren die Umfrageergebnisse noch deutlich schlechter: Lediglich 15 % der Teilnehmenden fühlten sich am Ende ihrer Einarbeitungszeit gewappnet für die Versorgung akuter Notfälle. Es konnte jedoch auch gezeigt werden, dass eine vollständige und strukturierte Einarbeitung zu einer Verbesserung des subjektiven Sicherheitsgefühls beitragen kann.

Stärker als in vielen anderen medizinischen Bereichen kommen in der Intensiv- und Notfallmedizin zahlreiche technische Geräte zum Einsatz, deren Anzahl aufgrund methodischer und technischer Fortschritte stetig zunimmt. Dabei besteht trotz eingeschränkter Datenlage Konsens darüber, dass von einer fehlerhaften Anwendung medizintechnischer Geräte ein hohes Risiko für die Patient:innensicherheit ausgehen kann und eine mangelhafte Einweisung Patient:innen sowie Anwender:innen gefährdet [2]. Daher schreibt das Medizinprodukterecht-Durchführungsgesetz (MPDG) eine Einweisung in die verwendeten Geräte vor [9, 18]. Es ist deshalb schockierend, dass lediglich 49 % der Teilnehmenden angaben, rechtskonform in die Geräte in ihrem Arbeitsbereich eingewiesen worden zu sein. Neben dem Gefährdungspotenzial und Sicherheitsrisiko für Patient:innen ist dies mit juristischen Haftungsrisiken bei der Anwendung und aufseiten der Betreibenden verbunden.

Als positiv ist zu bewerten, dass ein Großteil der Ärzt:innen berichtete, dass sie niederschwellig auf fach- oder oberärztliche Unterstützung zurückgreifen können. Eine derartige fach- oder oberärztliche Absicherung erhöhte auch relevant das subjektive Sicherheitsgefühl im Umgang mit planbaren Tätigkeiten.

Generell scheinen sich Assistenzärzt:innen zu Beginn ihres Einsatzes auf der Intensivstation oftmals nicht ausreichend vorbereitet zu fühlen, um den komplexen medizinischen Anforderungen gerecht zu werden [11, 13]. Dies wird auch durch den hohen Prozentsatz der Ärzt:innen deutlich, die zusätzlich zu ihrer Einarbeitung und Ausbildung im Krankenhaus außerklinische Fortbildungsangebote wie ICU-Beginner-Kurse o. Ä. wahrnehmen.

Dieses Gefühl der mangelnden fachlichen Kompetenz verursacht emotionalen Stress, einer der Hauptfaktoren für die hohe Prävalenz an Burn-out-Syndromen unter medizinischem Personal auf der Intensivstation [4, 7, 15]. Nahezu die Hälfte der Teilnehmenden unserer Umfrage gab an, sich durch die Arbeit psychisch belastet zu fühlen. Es zeigte sich jedoch auch, dass durch eine strukturierte Einarbeitung die psychische Belastung reduziert werden konnte.

Durch demografische Entwicklungen, medizinischen Fortschritt und zunehmende Technisierung wird der Bereich der Intensiv- und Notfallmedizin in den nächsten Jahren weiter an Bedeutung gewinnen. Parallel dazu wird auch der Bedarf an entsprechend qualifizierten Fachkräften steigen. Aus diesem Grund muss es ein vorrangiges Anliegen sein, die Arbeitsumgebung der Akut- und Intensivmedizin für junge Beschäftigte attraktiver zu gestalten. Von den Teilnehmenden unserer Umfrage konnten sich lediglich 35 % vorstellen, langfristig im intensiv- und notfallmedizinischen Sektor zu arbeiten. Da die Zufriedenheit am Arbeitsplatz zu großen Teilen von der subjektiv wahrgenommenen Qualität der Ausbildung abhängt [14, 17], ist die Güte der Ausbildung eine wichtige Stellschraube, damit junge Ärzt:innen und Pflegefachpersonen langfristig an den Bereich Intensiv- und Notfallmedizin gebunden werden.

Ein ausgereiftes Einarbeitungskonzept bringt somit auch ökonomische Vorteile mit sich, da es helfen kann, Einsatz- und Leistungsbereitschaft der Mitarbeitenden zu steigern und ungewollte Personalfluktuationen zu vermeiden [14].

Insbesondere vor dem Hintergrund des in deutschen Krankenhäusern herrschenden Fachkräftemangels gerät die auf Intensivstationen und in Notaufnahmen gängige Einarbeitungspraxis zunehmend in den Fokus des berufspolitischen Interesses, da ihr neben der Wissensvermittlung auch große Bedeutung bei der Rekrutierung junger Fachkräfte zukommt. So wurde im September 2023 eine weitere bundesweite Online-Umfrage veröffentlicht, die sich ebenfalls mit der Einarbeitung junger Fachkräfte auf deutschen Intensivstationen auseinandersetzt [8]. Die Urheberin dieser Befragung, die Junge DIVI, eine Initiative der Deutschen Interdisziplinären Vereinigung für Intensiv- und Notfallmedizin (DIVI), kommt in ihrer Untersuchung der Einarbeitungsqualität zu Ergebnissen, die den unseren in vielerlei Hinsicht ähneln bzw. sogar kongruent sind. Ähnlich zu unseren Resultaten gab in der DIVI-Umfrage von den befragten Ärzt:innen nur knapp ein Drittel an, über ein strukturiertes Einarbeitungskonzept zu verfügen (DIVI-Umfrage: 27 % vs. 36 %), wohingegen die Quote beim teilnehmenden Pflegefachpersonal mit immerhin fast 60 % genau wie in unserer Umfrage deutlich höher lag.

Weiterhin stellten die Autor:innen der DIVI-Umfrage ebenfalls fest, dass die angegebene Einarbeitungsdauer (insbesondere der ärztlichen Kolleg:innen) die von der DIVI empfohlenen drei Monate deutlich unterschritt. Besonders gravierend empfanden die Kolleg:innen der Jungen DIVI die Tatsache, dass sich am Ende der Einarbeitung fast die Hälfte aller Befragten nicht ausreichend gewappnet für die Tätigkeit auf der Intensivstation fühlte. Abschließend kommen beide Umfragen zum Ergebnis, dass sich nur ein Teil der Beschäftigten vorstellen kann, langfristig auf einer Intensivstation zu arbeiten [8].

Auch wenn die vorliegende Umfrage aufgrund einiger Limitationen, wie der unklaren Grundgesamtheit der Teilnehmenden, einer Heterogenität durch Berücksichtigung von Personal aus Notaufnahmen und von Intensivstationen sowie der Gefahr eines Selektionsbias, einer vorsichtigen und zurückhaltenden Interpretation bedarf, so kann dennoch ein oftmals defizitärer Status quo der Einarbeitung und Weiterbildung in der Intensiv- und Notfallmedizin erahnt werden. Diese Einschätzung wird durch die Ergebnisse anderer Arbeitsgruppen bekräftigt, die ebenfalls erhebliche Mängel an der gängigen Einarbeitungspraxis auf deutschen Intensivstationen konstatieren [8].

## Fazit

- Die Einarbeitung auf Intensivstationen und in Notaufnahmen ist in vielen Kliniken unvollständig, unstrukturiert und unzureichend:
  - Die Einarbeitungszeit verfehlte zumeist die Empfehlungen der DIVI [5].
  - Jeweils ca. 50 % der Befragten absolvierten nicht die vollständige Einarbeitungszeit, empfanden ihre Einarbeitung als unstrukturiert oder waren nicht rechtskonform in die Geräte ihres Arbeitsbereichs eingewiesen.
  - In der Folge bestand nach absolvierter Einarbeitung ein hohes Maß an Unsicherheit und persönlicher Belastung.
- Ca. 50 % hatten keine regelmäßigen Weiterbildungen und zudem mangelte es im Alltag oft an konstruktivem Feedback.
- Zur klinikübergreifenden Verbesserung der Einarbeitung bedarf es dringend strukturierter Konzepte.

## Supplementary Information

Zusatz-Abb. 4: Subjektives Sicherheitsgefühl bei planbaren Tätigkeiten und bei der Versorgung akuter Notfälle

Zusatz-Abb. 5: Psychische Belastung bei der Arbeit in der Notfall- und Intensivmedizin sowie persönliche Einschätzung der Pflegefachpersonen und Ärzt:innen, ob für sie eine langfristige Tätigkeit in diesem Arbeitsbereich vorstellbar wäre

Supplement: Fragen und Antwortmöglichkeiten des Online-Fragebogens zur Einarbeitung und Fortbildung auf Intensivstationen und in Notaufnahmen
