# Supplementary figures and images for: Onboarding in intensive care and emergency medicine in Germany
Source: Med Klin Intensivmed Notfmed. 2024 Feb 2;119(8):665–71. [Article in German] doi: 10.1007/s00063-024-01108-0 (PMC11538167; doi:10.1007/s00063-024-01108-0)

# Sicherheitsgefühl nach Einarbeitung

## Planbare Tätigkeiten

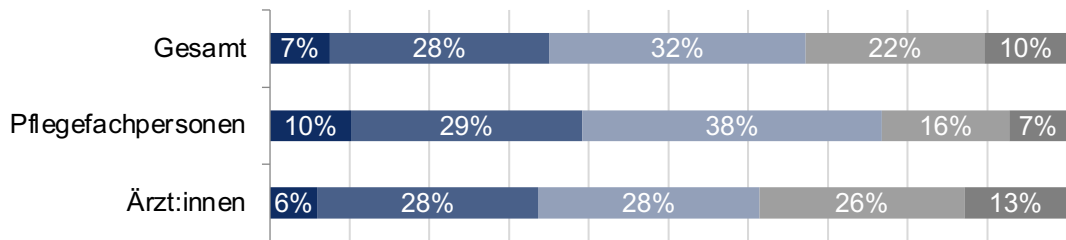

## Akute Notfälle

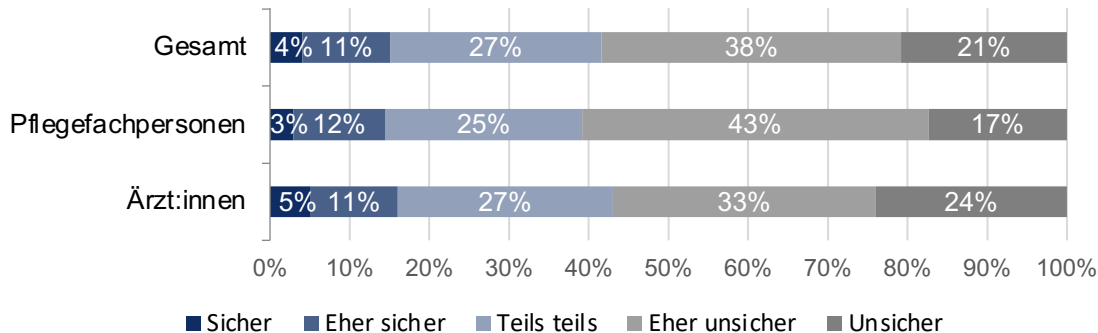

Supplement: Supplementary file 1 — Zusatz-Abb. 4: Subjektives Sicherheitsgefühl bei planbaren Tätigkeiten und bei der Versorgung akuter Notfälle [file 63_2024_1108_MOESM1_ESM.pdf]
