# Supplementary material for: Onboarding in intensive care and emergency medicine in Germany
Source: Med Klin Intensivmed Notfmed. 2024 Feb 2;119(8):665–71. [Article in German] doi: 10.1007/s00063-024-01108-0 (PMC11538167; doi:10.1007/s00063-024-01108-0)
Supplement: Supplementary file 2 — Zusatz-Abb. 5: Psychische Belastung bei der Arbeit in der Notfall- und Intensivmedizin sowie persönliche Einschätzung der Pflegefachpersonen und Ärzt:innen, ob für sie eine langfristige Tätigkeit in diesem Arbeitsbereich vorstellbar wäre [file 63_2024_1108_MOESM2_ESM.pdf]

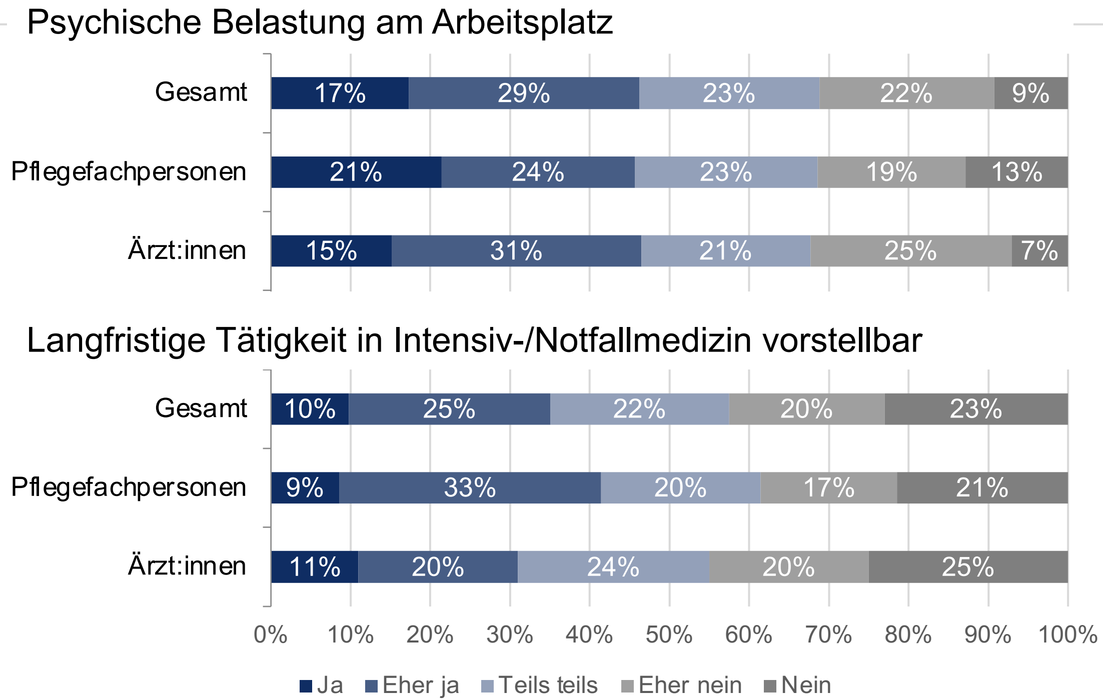
Psychische Belastung am Arbeitsplatz
Gesamt
17%
29%
23%
22%
9%
Pflegefachpersonen
21%
24%
23%
19%
13%
Ärzt:innen
15%
31%
21%
25%
7%
Langfristige Tätigkeit in Intensiv-/Notfallmedizin vorstellbar
Gesamt
10%
25%
22%
20%
23%
Pflegefachpersonen
9%
33%
20%
17%
21%
Ärzt:innen
11%
20%
24%
20%
25%
0%
10%
20%
30%
40%
50%
60%
70%
80%
90%
100%
Ja
Eher ja
Teils teils
Eher nein
Nein
