# Supplementary material for: Onboarding in intensive care and emergency medicine in Germany
Source: Med Klin Intensivmed Notfmed. 2024 Feb 2;119(8):665–71. [Article in German] doi: 10.1007/s00063-024-01108-0 (PMC11538167; doi:10.1007/s00063-024-01108-0)
Supplement: Supplementary file 3 — Supplement: Fragen und Antwortmöglichkeiten des Online-Fragebogens zur Einarbeitung und Fortbildung auf Intensivstationen und in Notaufnahmen [file 63_2024_1108_MOESM3_ESM.pdf]

Umfrage zur Einarbeitung und Lehre auf deutschen Intensivstationen

Liebe Kolleg*innen,

als Nachwuchsgesellschaft der Deutschen Gesellschaft für internistische Intensivmedizin und Notfallmedizin (DGIIN) möchten wir als Young DGIIN (YDGIIN) die Situation und Interessen der zukünftigen Generation von Intensivmediziner*innen, Intensivpfleger*innen und Notfallsanitäter*innen stärker in den Mittelpunkt rücken. Um bei fehlenden Daten eine Diskussionsgrundlage für die aktuelle Situation und etwaige Probleme zu schaffen, möchten wir den Status quo für junge Pflegenden in der Ausbildung/Fachweiterbildung und Assistenzärzt*innen auf deutschen Intensivstationen deskriptiv erfassen. Zu diesem Zweck haben wir einen Online-Fragebogen erstellt. Die Bearbeitungsdauer beträgt etwa 5-10min. Selbstverständlich sind alle Angaben anonymisiert und lassen keine Rückschlüsse auf die Teilnehmenden zu. Ebenso kann die Umfrage jederzeit abgebrochen werden. Nach Beendigung der Umfrage ist eine Vorstellung und Diskussion der Ergebnisse auf der DGIIN Jahrestagung 2023 geplant, zu der wir bereits jetzt herzlich einladen wollen

Vielen Dank für die Unterstützung!

Ihre/Eure YDGIIN

"Ich weiß, dass die Teilnahme an dieser Studie freiwillig ist und ich meine Teilnahme ohne Angabe von Gründen jederzeit beenden kann. Hiermit erkläre ich mich damit einverstanden, dass meine Angaben für die o.g. wissenschaftlichen Zwecke verwendet werden dürfen. Ich bin darüber informiert, dass es aufgrund der Anonymität der Umfrage nicht möglich ist, nachträglich individuelle Datensätze von der Verarbeitung auszuschließen, zu korrigieren, zu löschen oder zu exportieren."

Demographische Daten

1. Bitte geben Sie Ihr Geschlecht an

(Einfachauswahl)

- weiblich
- männlich
- divers

2. Wie viele Jahre Berufserfahrung haben Sie?

(Slider [Jahre])

3. Welcher Berufsgruppe gehören Sie an?

(Einfachauswahl)

- Ärztliches Personal
- Pflegepersonal
- Rettungsdienst
- Sonstiges

4.A.1 Welchen Ausbildungsgrad haben Sie? (Mehrfachantworten möglich)

Wenn Berufsgruppe = Pflege
(Mehrfachauswahl)

- 3-jährige Ausbildung mit staatlichen Examen Pflegefachfrau / Pflegefachmann
- Fachweiterbildung Anästhesie und Intensiv
- Fachweiterbildung Notfallpflege
- Bachelor of Arts/ Science
- Master of Arts/ Science
- Sonstiges

4.A.2 Haben Sie eine ergänzende Weiterbildung oder ein ergänzendes Studium? (Mehrfachauswahl möglich)

Wenn Berufsgruppe = Pflege
(Mehrfachauswahl)

- Pflegewissenschaft
- Pflegemanagement
- Pflegepädagogik
- Advanced Practice Nursing
- Ich befinde mich gerade in einem Studiengang bzw. einer Weiterbildung
- Sonstiges:
- Nein

4.A.3 Welche Funktion haben Sie?

Wenn Berufsgruppe = Pflege
(Einfachauswahl)

- Pflegefachfrau/ Pflegefachmann
- Praxisanleiter*in
- Stationsleitung
- Advanced practice nurse (APN)
- Sonstiges

4.B.1 Welchen Ausbildungsgrad haben Sie? (Mehrfachantworten möglich)

Wenn Berufsgruppe = Rettungsdienst
(Mehrfachauswahl)

- Rettungsassistent*in
- Notfallsanitäter*in
- Rettungshelfer*in
- Sonstiges:

4.C.1 Welche Funktion haben Sie?

Berufsgruppe = Medizin
(Einfachauswahl)

- Assistenzärzt*in
- Fachärzt*in
- (Funktions-)Oberärzt*in

4.C.2 Welche Zusatzweiterbildung haben Sie? (Mehrfachauswahl möglich)

Berufsgruppe = Medizin
(Einfachauswahl)

- Intensivmedizin
- Notfallmedizin
- Sonstige
- Keine

5. In welchen Bereich (Krankenhaus/ Notfallmedizin) arbeiten Sie aktuell hauptsächlich?

(Einfachauswahl)

- Intensivstation / IMC
- Notaufnahme
- PACU / Aufwachraum / Anästhesie
- Rettungsdienst
- Sonstiges:

6. Welcher medizinischen Fachrichtung ist Ihr Arbeitsbereich hauptsächlich zugeordnet?

(Einfachauswahl)

- Innere Medizin
- Anästhesie
- Operative Medizin
- Notfallmedizin
- Pädiatrie/ Neonatologie
- Intensivmedizin
- Sonstiges

7. Wie viele Jahre an Berufserfahrung (ohne Ausbildungszeit) haben Sie in der Intensiv- und Notfallmedizin?

(Slider [Jahre])

8. Wie viele Betten hat Ihr Krankenhaus insgesamt?

(Slider [10-4000, 10er Schritte])

9. Wer ist Träger Ihres Krankenhauses in dem Sie hauptsächlich arbeiten?

(Erklärung siehe unter Antwortmöglichkeiten)

(Einfachauswahl)

- Öffentliche Krankenhausträger
- Freigemeinnütziger Krankenhausträger
- Privater Krankenhausträger
- Privatklinik

Öffentlicher Krankenhausträger

- *Bund*
- *Länder*
- *Bezirke*
- *Landkreise*
- *Gemeinden*
- *Kommunale Gebietskörperschaften*
- *Städte*
- *Öffentliche Zweckverbände*

- *Gesellschaften mit beschränkter Haftung (GmbH) ⇒ nur dann in öffentlicher Trägerschaft, wenn Gebietskörperschaften (Bund, Länder, ...), Zusammenschlüsse solcher Körperschaften oder Sozialversicherungsträger (z. B. Rentenversicherungsträger) mehr als 50 % des Nennkapitals oder Stimmrechts halten!*

Freigemeinnütziger Krankenhausträger

- *Gemeinnützige Vereine und Stiftungen*
- *Karitative Organisationen*
- *Kirchliche Orden*
- *Kongregationen (Klosterverbände)*

Privater Krankenhausträger

- *Aktiengesellschaften (AG)*
- *Gesellschaften bürgerlichen Rechts (GbR)*
- *Gesellschaften mit beschränkter Haftung (GmbH)*
- *Rechtsfähige Vereine*
- *Stiftungen des Privatrechts*

Privatklinik

- *Privatkliniken sind von Krankenhäusern in privater Trägerschaft abzugrenzen. Im Gegensatz zu solchen besitzen sie keine Zulassung zur Abrechnung ihrer Leistungen über die Gesetzliche Krankenversicherung und sind somit nicht zur Teilnahme an der gesetzlichen Gesundheitsversorgung berechtigt. Sie sind folglich nur für die Versorgung von Privatpatienten zuständig.*

10. Arbeiten Sie an einem Universitätsklinikum?

(Ja/Nein Frage)

11. Arbeiten Sie an einem Krankenhaus der Maximalversorgung?

(Ja/Nein Frage)

12.In welchem Bundesland liegt das Krankenhaus in dem Sie arbeiten?

(Einfachauswahl)

- Baden Württemberg
- Bayern
- Berlin
- Brandenburg
- Bremen

- Hamburg
- Hessen
- Mecklenburg-Vorpommern
- Niedersachsen
- Nordrhein-Westfalen
- Rheinland-Pfalz
- Saarland
- Sachsen-Anhalt
- Sachsen
- Schleswig-Holstein
- Thüringen

12. Falls Sie auf einer Intensivstation arbeiten, wie viele Intensivbetten hat Ihre Station insgesamt?

(Slider [Anzahl Intensivbetten])

Besetzung Dienst

13. Wie lange sind die regulären Dienste auf Intensivstation / in der ZNA / im Rettungsdienst? (Angabe in Stunden)

(Slider [Stunden])

14. Wie viele Intensiv-Patient*in sind bei regelhafter Besetzung pro Kolleg*in tagsüber eingeplant?

(Slider [1-30])

15. Ist im Regelfall rund um die Uhr mindestens ein/e Fach*ärztin auf der Intensivstation im Dienst eingeplant?

(Ja/Nein Frage)

16. Ist jederzeit ein/e Oberärzt/in erreichbar?

(Einfachauswahl)

- Ja, mit intensivmedizinischer Erfahrung

- Ja, (z.T.) ohne intensivmedizinische Erfahrung
- Nein

17. Haben Sie das Gefühl Ihren oberärztlichen Hintergrund bei Unsicherheiten oder Notfällen jederzeit kontaktieren zu können?

(Ja/Nein Frage)

Einarbeitung ÄD

18. Wie viele Tage waren für die Einarbeitung auf der Intensivstation/ZNA eingeplant?

(Slider [Tage])

19. Haben Sie die vollständig geplante Einarbeitung absolviert?

(Ja/Nein Frage])

20. Wie war der geschätzte prozentuale Anteil folgender Unterpunkte während ihrer Einarbeitung?

(Slider [Summe 100%])

- Theoretische Inhalte (Seminare, Vorträge, Vorlesung)
- Praktische Inhalte als Simulation (an Puppen, SkillsLab, etc)
- Praktische Inhalte an Patient*innen unter Anleitung
- Unmittelbare Regelversorgung von Patient*innen

21. Wurden Sie im Vorfeld ihrer Einarbeitung / Einsatzbeginn auf der Intensivstation / ZNA über mögliche externe Inhalte (DGIIN/DIVI Kurs, Sonographie Kurse, o.ä.) durch ihre Klinik informiert?

(Ja/Nein Frage)

22. Haben Sie an externen Kursen teilgenommen?

(Mehrfachauswahl)

- ICU Beginner Kurs
- Notarzt Kurs
- Sonographie Kurs
- Echokardiographie Kurs
- ECMO Kurs
- Sonstiges:

23. Wurden die Kosten für die Kurse durch ihre Klinik erstattet?

(Ja/Nein Frage + Keine Antwort)

24. Gibt es ein strukturiertes Einarbeitungskonzept inklusive Skript?

- Strukturierte Einarbeitung mit Skript
- Strukturierte Einarbeitung ohne Skript
- Unstrukturierte Einarbeitung mit Skript
- Unstrukturierte Einarbeitung, kein Skript

25. Sind Sie in alle Geräte, die auf der Intensivstation durch Sie bedient werden, strukturiert eingewiesen worden?

(Ja/Nein Frage)

26. Sind Sie in alle Geräte, die auf der Intensivstation durch Sie bedient werden, offiziell eingewiesen worden?

(Ja/Nein Frage)

27. Wie sicher fühlten Sie sich in der Benutzung aller Geräte nach der Einarbeitung?

(Slider 1-5, sehr sicher/unsicher, eher sicher/unsicher, ausreichend)

28. Werden die ersten Dienste nach Einarbeitung mit zusätzlicher Besetzung geplant? („Mitlaufdienst“)

(Ja/Nein Frage)

29. Wenn ja, ab dem wievielten Dienst nach Einarbeitung ist man „alleine“ eingeteilt?

(Slider Anzahl Dienste/Arbeitstage)

30. Finden regelmäßig Fortbildung im Bereich der Intensiv- und Notfallmedizin statt?

(Ja/Nein Frage)

31. Wenn ja, wie oft?

(Einfachauswahl)

- Wöchentlich
- Zweiwöchentlich
- Monatlich
- Seltener

32. Finden regelmäßig interdisziplinäre Praxisübungen mit dem Training von Notfallsituationen statt?

(Ja/Nein Frage)

33. Skalierungs-Fragen

(Skala 1-5, Ich stimme (eher) zu – teils – ich stimme (eher) nicht zu)

- Ich habe mich in meinen ersten Diensten nach der Einarbeitung sicher im Umgang mit den „planbaren“ Tätigkeiten gefühlt
- Ich habe mich in meinen ersten Diensten nach der Einarbeitung sicher in der Versorgung von akuten Notfällen gefühlt
- Ich hatte 24h die Chance mir unkompliziert Hilfe durch FA/FÄ oder OA/OÄ zu holen
- Ich habe mich nach der Einarbeitung gut in das Team der Intensivstation integriert gefühlt
- Ich bekomme regelmäßig Feedback von meinen Vorgesetzten
- Es erfolgt eine regelmäßige Nachbesprechung nach Notfällen
- Ich fühle mich durch die Arbeit psychisch belastet
- Ich könnte mir vorstellen langfristig in diesem Bereich zu arbeiten

Mitarbeit

Haben Sie Interesse bei der Young DGIIN mitzuarbeiten/ teilzunehmen, um Ihre Ideen und Erfahrungen einzubringen?

Bitte wählen Sie nur eine der folgenden Antworten aus:

- Ja
- Nein

Wenn Sie Interesse haben, können Sie entweder hier Ihre Mailadresse eintragen oder eine Mail an die Geschäftsstelle der DGIIN (gs@dgiin.de) schicken.

Durch die Angabe Ihrer Mailadresse, kann kein Zusammenhang zu Ihren vorherigen Antworten hergestellt werden!

Bitte geben Sie Ihre Antwort hier ein:

Vielen Dank, dass Sie an der Umfrage teilgenommen haben.

Wir würden uns über Mitarbeit freuen.

Ihre / Eure YDGIIN

Übermittlung Ihres ausgefüllten Fragebogens:
Vielen Dank für die Beantwortung des Fragebogens.
